# Non-Adaptive Methods for Fetal ECG Signal Processing: A Review and Appraisal

**DOI:** 10.3390/s18113648

**Published:** 2018-10-27

**Authors:** Rene Jaros, Radek Martinek, Radana Kahankova

**Affiliations:** Department of Cybernetics and Biomedical Engineering, Faculty of Electrical Engineering and Computer Science, VSB—Technical University of Ostrava, 17. listopadu 15, 708 33 Ostrava, Czech Republic; radana.kahankova@vsb.cz

**Keywords:** non-adaptive filtering, fetal electrocardiogram extraction, fetal monitoring, digital signal processing

## Abstract

Fetal electrocardiography is among the most promising methods of modern electronic fetal monitoring. However, before they can be fully deployed in the clinical practice as a gold standard, the challenges associated with the signal quality must be solved. During the last two decades, a great amount of articles dealing with improving the quality of the fetal electrocardiogram signal acquired from the abdominal recordings have been introduced. This article aims to present an extensive literature survey of different non-adaptive signal processing methods applied for fetal electrocardiogram extraction and enhancement. It is limiting that a different non-adaptive method works well for each type of signal, but independent component analysis, principal component analysis and wavelet transforms are the most commonly published methods of signal processing and have good accuracy and speed of algorithms.

## 1. Introduction

Fetal monitoring during pregnancy is very important for identifying many factors that may negatively affect the health of the fetus, may prevent intrauterine death (most commonly occurring in the home environment), or permanent damage to the fetus [1,2,3,4,5]. These factors may not only be harmful to the health of the fetus, but also to the health of the mother. Some issues, such as premature birth, hypoxia, or intrauterine retardation, are dangerous both for the fetus and for the mother. Fetal monitoring includes methods such as fetal electrocardiography (fECG) [1,2,3,6,7,8,9,10,11], fetal phonocardiography (fPCG) [4,5,12,13,14,15,16,17,18,19,20,21], fetal echocardiography (fECHO) [22,23,24,25], fetal magnetocardiography (fMCG) [26,27,28,29,30], and cardiotocography (CTG) [9,31,32,33], which is based on Doppler ultrasound. Each fetal monitoring method has its advantages as well as disadvantages, and it should be emphasized that, regarding long-term measurements, a 20-min measurement is relatively short in order to obtain real information about the condition of the fetus.

This field also attracts the attention of researchers focused on signal processing and noise removal, especially in the last 10 years. This is also related to the purpose of this work, which is to describe the processing of the fECG signal by many different non-adaptive methods. Description and comparison of these methods could produce useful material for those that are trying to find appropriate non-adaptive processing methods without going through a constantly increasing amount of research articles on this topic.

**Figure 1:**
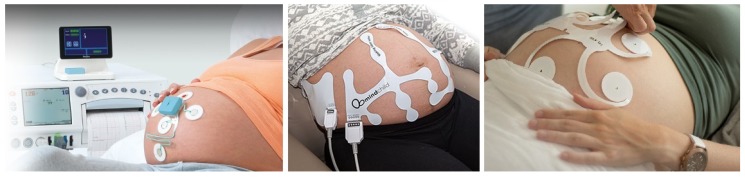
Novii Wireless Patch System (**left**), MERIDIAN M110 Fetal Monitoring System (**middle**), and The Nemo Fetal Monitoring System (**right**).

Figure 1 shows the main commercially available devices for non-invasive fECG measurement from three different companies: Monica Healthcare (Nottingham, UK) (Novii Wireless Patch System), MindChild Medical (North Andover, MA, USA) (MERIDIAN M110 Fetal Monitoring System), and Nemo Healthcare (Veldhoven, the Netherlands) (The Nemo Fetal Monitoring System). The recently growing amount of sophisticated fECG-based devices for fetal monitoring only confirms that there is a gradual upturn from conventional electronic fetal monitoring (EFM) using CTG to fECG [34,35,36].

**Figure 2:**
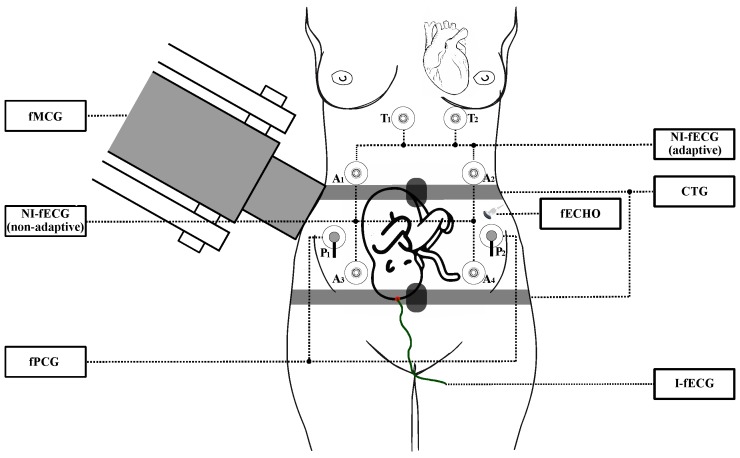
Fetal monitoring and signal processing techniques.

Figure 2 shows various fetal monitoring techniques described, such as fMCG, CTG, invasive fECG (I-fECG), non-invasive fECG (NI-fECG), and fPCG. The figure shows the difference between the use of electrodes in the adaptive processing of the fECG signal, where the electrodes from the thoracic area (marked in the figure as $T_{1}$ and $T_{2}$) and the abdominal area (marked in the figure as $A_{1}$, $A_{2}$, $A_{3}$ and $A_{4}$), and between the use of electrodes in the non-adaptive processing of the fECG signal where only the abdominal electrode needs to be used. It is clear that the maternal heart signal is extending from the thoracic area to the abdominal area of the pregnant woman, so signal measured in the abdominal area is composed of a maternal fetal component. The ECG signal measured on the pregnant woman’s chest is considered to be a pure maternal ECG (mECG) signal because it theoretically does not contain the fetal component. Furthermore, the picture shows the possibility of measuring fPCG using microphones attached to the mother’s abdomen (in the picture, the microphones are marked as $P_{1}$ and $P_{2}$).

Each of the above mentioned EFM methods has advantages and disadvantages. In Table 1, we summarize the main information about each of the techniques showed in Figure 2, such as the technical solution or the limitation of its usage in terms of gestation age. Moreover, Table 1 offers comparison of the most significant benefits and drawbacks of the EFM methods [1,2,4,16,37,38,39]. The summary implies that NI-fECG is a very promising method. This approach is very safe, simple and cheap, and, based on some recent studies [40,41], fECG achieves better results than the conventional CTG. Additionally, it also appears to be a more accurate method to be used with patients with higher body mass index (BMI).

### 1.1. Fetal Electrocardiography

Fetal ECG is a diagnostic method that captures the electrical activity of the fetal heart muscle. This method is very important for the detection of cardiac arrhythmias, ischemia and other heart abnormalities [3]. Fetal ECG measurement is performed either invasively, using the transvaginal scalp electrode, or non-invasively, by means of electrodes by placed in various areas of the mother’s abdomen. The invasive approach provides a very good-quality recording due to the direct contact of the electrode with the fetal head, but it also has a number of disadvantages, such as the possibility of passing infection into the mother or fetus body [2]. When the contact of the electrode with the fetal head is insufficient, this results in poor signal transmission and the isolines fluctuate. On the other hand, the non-invasive approach is not burdensome for the mother or the fetus and can be used even during the delivery, but the signal needs to be processed properly in order to obtain the appropriate form of fECG because it is contaminated by the maternal component and the noise, including biological artifacts (maternal and fetal movements, maternal and fetal breathing, maternal and fetal muscle activity, uterine contractions, etc.) as well as technical artifacts (electrostatic potentials, network interference, etc.) [1,2].

Fetal ECG and mECG can be measured in a non-invasive manner, but the limitations include a very low value of signal-to-noise ratio (SNR), the need for proper electrode positioning, the need for an optimal number of electrodes because each electrode carries its own noise, and that fECG is often masked by the mECG signal, power line noise, maternal electromyogram, etc. Fetal ECG is a passive measurement that requires much more comprehensive signal processing than is the case with CTG, but it is less costly [16]. When comparing fECG with ultrasound, it provides much more information about heart defects because these defects have specific electrical manifestations.

**Figure 3:**
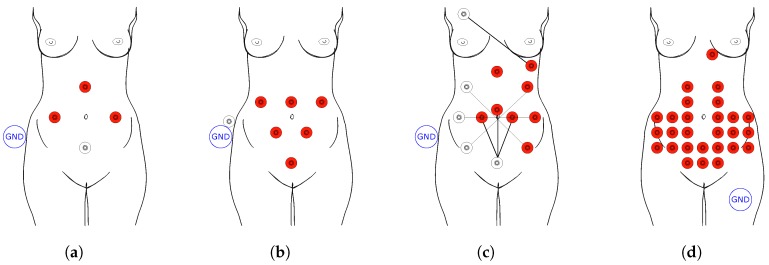
An example of positioning of the electrodes during fetal electrocardiography. Red electrode is the active electrode, white electrode is the reference electrode and ground (GND) represents ground electrode. (**a**) five electrodes; (**b**) eight electrodes; (**c**) 14 electrodes; (**d**) 28 electrodes.

Currently, the main objective of fECG is to determine fetal heart rate (fHR), and since the position of the electrodes in the abdominal region of the pregnant woman is not standardized due to the different fetal position, and hence the fetal heart, the automatic determination of fHR is a major problem. Figure 3 shows examples of positioning of the electrodes in the abdominal region of the mother for measuring the abdominal electrocardiography signal (aECG) [7,44,45]. The most important step is to correctly remove the noise as well as the maternal components. Typically, the fHR is about 120 to 160 beats per minute (bpm) and maternal heart rate (mHR) about 70 to 80 bpm [6]. Although there is no direct connection between the mother and the fetus, the hormones and the placenta can greatly influence fHR and fetal pressure. The fECG and mECG amplitudes also differ considerably, wherein the mECG amplitude is several times greater than the fECG amplitude [2]. Very often, the QRS complex of the mother (mQRS) and the QRS complex of the fetus (fQRS) overlap, and since the mECG amplitude is bigger, it is very difficult to determine whether the fetal heart beat occurred at the particular place of overlapping.

**Figure 4:**
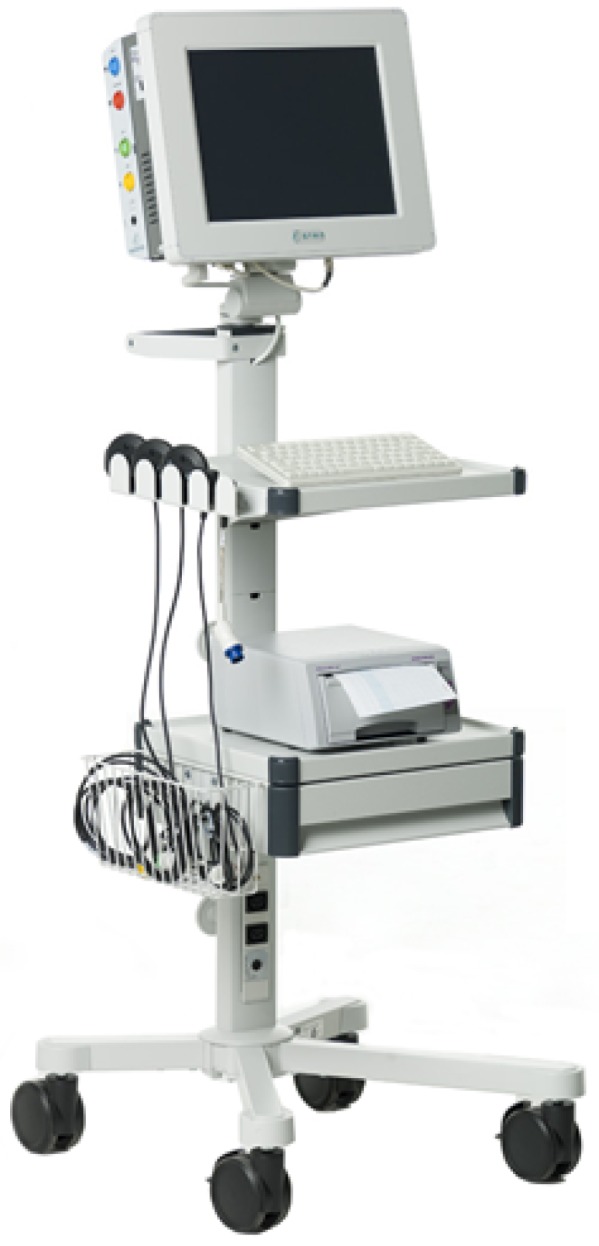
The S31 ST segment analyzer.

ST (interval between S and T waves on the fECG waveform) segment analysis of the fECG obtained has a great prospect for fetal monitoring. This analysis was designed to provide objective fetal status information following fHR monitoring. Myocardial cell repolarization is very sensitive to hypoxia-induced metabolic dysfunction, which is reflected in ECG waveform changes, such as an increase in the ST segment and T waves. Therefore, ST segment analysis monitors the ability of the myocardium to respond to hypoxia, during which there is an increase in the ST segment and T waves. A change in the ratio of ST segment analysis is considered a change in cellular ionic fluxes during anaerobic metabolism in the heart [3]. If hypoxia continues to deepen, the increase in T wave is getting bigger and bigger until a large T wave drop occurs, resulting in a high risk of acute cardiovascular failure. Combined fHR and ST segment analysis monitoring reduces incorrect cases of premature termination of pregnancy. This analysis can be reliably performed even in the absence of an ECG vector, and it has been proved that the use of ST segment analysis leads to early identification of cases where acidosis (a decrease in the concentration of standard bicarbonates below the reference value) occurs during the delivery. The drawback of ST analysis is its invasiveness and its limited usage. The signal is recorded using an electrode placed directly on fetal scalp so it can be only performed during the delivery, after the rupture of the membrane with amniotic fluid. This technique is enormously influenced by noise and is focused only on issues that affect the heart as a whole. Hence, the issues affecting another part of the heart will not be detected. Equation $ST_{\mathrm{analysis}}=\frac{T}{QRS}$ describes the calculation of this important parameter for fetal status monitoring, where T is the T wave amplitude and QRS is the QRS complex amplitude [8]. Figure 4 shows the classic S31 device for ST segment analysis [46].

**Figure 5:**
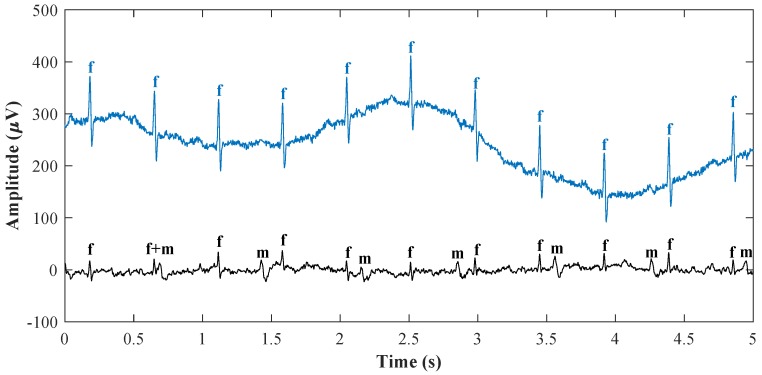
ECG waveforms measured by the scalp electrode (**upper**) and the abdominal electrode (**lower**).

**Figure 6:**
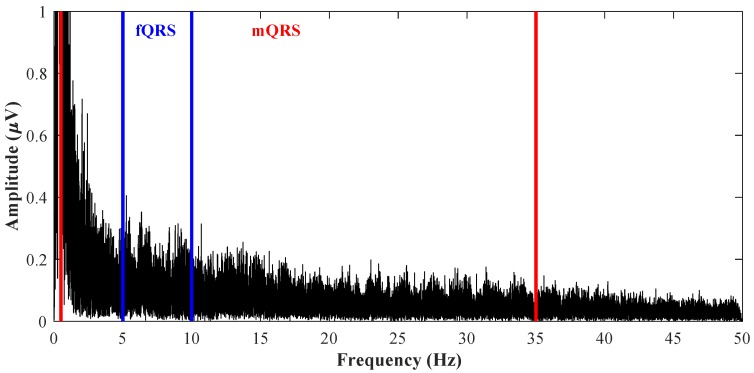
The spectrum of the aECG signal. Blue vertical lines represent a frequency band of fQRS complex and red vertical lines represent a frequency band of mQRS complex.

QRS complexes overlap can be tracked from the waveforms shown in Figure 5, where the upper signal is the reference ideal fECG measured by the scalp electrode and the lower signal is the aECG signal measured by the abdominal electrode in the mother’s abdominal region. In this figure, letter *f* denotes fQRS complexes, and letter *m* denotes mQRS complexes. QRS complexes overlap can be seen also in Figure 6 showing the aECG waveform spectrum from Figure 5. It can be seen that the frequency domain of fQRS complexes, lying approximately in the range from 10 to 15 Hz and marked in blue colour, overlaps with the frequency domain of mQRS complexes, lying approximately in the range from 0.5 to 35 Hz and marked in red colour, and it can be seen that ordinary filtration of a certain band cannot be used for the extraction of fECG. The values of the frequency bands of fQRS complexes and mQRS complexes were obtained from the study conducted by Sameni et al. in 2010 [3]. Figure 5 and Figure 6 were created from real data from the abdominal and direct fECG database (adfecgdb) [10,47,48,49,50], which contains five records from different women that are 5 min long with a sampling frequency of 1 kHz and with 16-bit resolution.

### 1.2. Basic Distribution of Signal Processing Methods

Some signal processing methods can be used in many other areas, than only in fECG extraction, such as fPCG, electroencephalography, voice recognition, image identification, etc. The basic methods of fECG signal processing can be divided into adaptive and non-adaptive ones. Both types of methods have their advantages and disadvantages and are becoming more and more refined.

Adaptive methods of signal processing are based on a learning system. In fECG signal processing, these methods require a clean mECG signal from the mother’s chest and an aECG signal, which contains both maternal and fetal components and noises. Hybrid neural network (HNN), artificial neural networks (ANN), and techniques of adaptive neuro-fuzzy inference system (ANFIS) fall into nonlinear adaptive techniques and the methods based on the theory of Kalman filtering (KF), least mean squares algorithm (LMS), recursive least squares algorithm (RLS), and methods based on adaptive linear neuron (ADALINE) [1,2,3,6] fall into linear adaptive methods.

The non-adaptive methods do not use any adaptive system for signal processing and only work with one or more records that contain components that need to be processed. Thus, in fECG signal processing, these methods do not need electrodes placed at the maternal chest as with adaptive methods and only use electrodes placed at the maternal abdomen. The non-adaptive methods have constant coefficient values and the system does not adapt to the existing circumstances but performs work for which it is intended [2]. These methods are very fast and accurate, but their disadvantage is that they are time invariant in nature compared with the adaptive methods. For interference elimination in aECG signal, the non-adaptive methods use either single channel signal sources, which are implemented by many methodologies, or multichannel signal sources, which are processed by blind source separation techniques (BSS) [1,2,3,6].

In this paper, Section 2 deals with non-adaptive methods that use single channel signal sources for extraction of fECG signal. Then, Section 3 deals with non-adaptive methods that use multichannel signal sources for extraction of fECG signal. Section 4 shows the possibility of creation hybrid algorithms, in Section 5, discussion is provided, and, in Section 6, conclusions are provided.

## 2. Single Channel Signal Sources

Many methods use only single channel signal sources. This group of methods includes methods based on wavelet transform (WT), correlation technique (CT), subtraction technique (ST), averaging technique (AT), filtering techniques (FT) such as finite impulse response (FIR) and infinite impulse response (IIR) filtering, Wiener filtering (WF), fixed filtering such as low-pass filtering (LPF) and high-pass filtering (HPF), de-shape short time Fourier transform (STFT) and nonlocal median (NM), single channel BSS (SCBSS), template subtraction (TS), sequential total variation denoising (STVD), empirical mode decomposition (EMD), etc. All of these methods will be discussed below. Table 2, at the end of Section 2, shows a comparison of the different single channel methods.

### 2.1. Wavelet Transform

There are many types of wavelet transform such as discrete wavelet transform (DWT), complex wavelet transform (CWT), pitch synchronous wavelet transform (PSWT), etc., and the choice depends on the application and the type of the input signal [51]. These methods provide information in time and frequency domains, so they are very effective in non-stationary signals or multiple component signals. Wavelet transform is basically a convolution operation of the signal and wavelet function [52]. This method decomposes a signal into a detail signal, which contains the upper half of frequency components, and into an approximation signal, which contains the lower half of frequency. This decomposition can be performed again on the approximation signal and then will create the second detail and approximation signal. They are also used for creation of a hybrid method as preprocessing [5].

Hassanpour et al. [51] used DWT for estimation of fECG. Their algorithm consisted of two steps. In the first one, they used a two-level WT to extract fECG and mECG and, in the second one, they used a Savitzky–Golay smoothing filter (SGSF) on fECG signal to attenuate the effect of the noise. This filter is a LPF, well adapted for smoothing noisy data. The type of wavelet of DWT is the same in shape as the heart beat wave and its energy spectrum is around low frequencies. They used three pieces of synthetics data, where mECG were ten times stronger than the energy of fECG, and real data from the database developed by De-Moor [53], which were 10 s long and with a 250 Hz sampling rate. They concluded that this method has promising results for fECG extraction.

Bhoker et al. [52] used DWT for extraction of fECG and then for detection of R-peaks. In the first step, they used WT for decomposing into fECG and mECG and, in the second step, the fetal R-peaks are detected from the extracted fECG signal. For estimation, they used the data from physionet NI-fECG database [54,55], which were 10 s long and with a sampling rate of 1 kHz with 16-bit resolution. They used 15 different signals containing two thoracic and three to four abdominal signals filtered by a 50 Hz notch filter. The estimation of fECG was conducted by subtraction of the extracted mECG from aECG because the energy of mECG in aECG was much higher than the energy of fECG, and DWT better estimated mECG signal than fECG. They came to the conclusion that DWT successfully detected the same number of R-peaks in fECG as in the original aECG.

Karvounis et al. [56] used CWT for automation extraction of fHR. This method is very successful in detection of the changing properties of non-stationary signals. The algorithm of CWT is performed in four stages. The first one is preprocessing through signal averaging, the second one is identification of mother heart beats, the third one is identification of fetal heart beats, including false positive, and in the fourth one a heuristic algorithm is applied to find out the overlapped fQRS points and to remove incorrectly detected QRS. They used 15 recordings from the fECG database of the University of Nottingham [57]; these recordings are 1 min long with three measurement locations on the abdomen, sampling frequency of 300 Hz and with 12-bit resolution. They came to the conclusion that, from the total of 1975 beats, the CWT method detected 1954 correctly, 12 were missed and nine misdetected. However, most of the wrongly detected QRS points were located at the starting and ending segment of the signals. This method was recognized as very efficient; it improves the analysis of the signal due to the information from the imaginary part of the wavelets and it is able to extract mHR for parallel monitoring.

Kumar et al. [58] introduced a new fECG extraction method using two passes PSWT. This method is based on a concept, which can capture period to period fluctuations of the signal. The first one is estimated mECG from a corrupted aECG signal by pitch synchronous decomposition and then recovers the desired fECG. They marked PSWT as very accurate and useful especially in a low fECG power circumstance. Minimal information loss is provided when detecting fECG and mECG. By performing PSWT, SNR was improved and the extracted signals could be used to determine the fHR.

### 2.2. Correlation Technique

Auto and cross correlation techniques find similarities between two samples of a given signal as a function of time lag and thanks to that, CT emphasizes periodically occurring correlated wavelets by highlighting non-periodic uncorrelated events (noise). Correlation technique is used for fECG extraction, but only sometimes managed to detect fECG signal well and, due to deficiency of proper template wanted, it fails to detect the accurate signal [57]. In fECG extraction, the templates depend on many factors, such as gestational age, BMI, number of fetuses, the mother’s age, etc. This method is also bad in detection of multiple fetus monitoring and not effective in the detection of non-stationary signals. Correlation technique obtains an averaged fECG signal by using a suitable correlation function, which is subtracted from aECG signal in order to obtain fECG signal from the two signals via mECG signal and input aECG signal.

Bemmel [59] shows a method which includes auto correlation and cross correlation techniques for the detection of weak fECG signal from maternal aECG. He used synthetic and real data for the evaluation and came to the conclusion that the extracted fECG is effective but not efficient enough.

### 2.3. Subtraction Technique

It is the oldest, simplest and straightforward approach of signal processing. Today, it is an obsolete method due to mismatching mECG signal with aECG signal. In fECG extraction, mECG is very dominant, so this technique fails to remove mECG signal and, due to improper matching instead of removal, the mECG is summed up with the abdominal signal and it is impossible to detect a pure fECG signal. This method works by sorting an aECG signal and mECG signal in such a way that mECG signal can be subtracted from aECG signal and the resulting signal is fECG signal with some residual noise, which is eliminated in the next step.

Bergveld et al. [60] used the ST method for suppression of an mECG signal from aECG signal in order to obtain fECG signal. They used real and synthetic data for the experiments and concluded that pure fECG is hardly yielded when signals are being subtracted because the mECG measured from thorax is not mostly the same as the mECG measured from the abdomen.

Levkov et al. [61] dealt with improving the ST method. They used hardware measurement and they monitored the interference period of software measurement to compensate frequency deviation. The filter module was introduced into the algorithm in order to increase the flexibility of the construction. They used digital filtering on linear segments of the signal to remove the interference components. They concluded that the efficiency of the method is not dependent on the amplitude and frequency of the interference and that the main advantage of the ST method is that it, virtually, completely eliminates the network interference from fECG signal without breaking its spectrum as it occurs in other methods. For this evaluation, they used two signals, wherein the first one was taken from their own database called ‘clean’ and the other one was a synthetized signal.

### 2.4. Averaging Technique

This method is one of the most used methods in the last century for extraction of fECG signal by using only one abdominal lead. Maternal R waves have large amplitude, so they are easy to detect by threshold detectors from aECG signal. The reference signal, which is the same as the interval of mECG signal, is obtained by averaging the succeeding intervals of aECG signals containing mQRS complex in the equal phase. In the next step, fECG and interferences are suppressed from reference due to the fact that they are statistically independent from mECG signal and then, by subtraction of the reference from aECG signal, a signal completely without mECG is created [2]. Averaging technique were used for de-noising or signal extraction and they improve SNR, but AT will be inaccurate if the relative time position from one segment to the other is not synchronized [5].

Hen et al. [62] have dealt with the AT method and have found that the limitation of the algorithm is a necessity to artificially convert a non-periodic signal to a regular one. They came to the conclusion that the AT method improves SNR by 10 to 20 decibels (dB), so that it is then possible to extract fECG which shows fetal P and T waves, as well as baseline changes during labor and delivery.

### 2.5. Filtering Methodologies

Abdominal ECG from pregnant women contains a fetal component, which is important for us, and artefacts such as a maternal component and noise. These artefacts can be filtered by using a large number of methods, e.g., FIR filters, IIR filters, WF, projective filtering (PF), linear time domain filters, frequency filters and fixed filters. Some types of artefacts can be filtered directly in the time domain because the spectral characteristics may not be required, and, in most cases, it is faster than frequency domain filtering [2]. In cases where the spectrum of the signal and noise is overlapping, linear filters are insufficient. Another possibility is using synchronized signal averaging or a moving window averaging filter. The reliability of the parameter or signal extraction based on conventional filtering depends solely on the information from either the frequency domain or power spectral density. Varady [63] concluded that even conventional filtering seems to improve SNR by removing most of the out of band noise, where, in the in-band noise, it continues to exist. Frequency domain filters contain low pass, high pass, band pass and notch filtering features [2,5].

Alcaraz et al. [64] used an HPF with 0.5 Hz cut off frequency for baseline wandering elimination, an LPF with 70 Hz cut off frequency for high frequency elimination and a notch filter for power line interference elimination. They used it as preprocessing with 20 real-time ECG recordings, which are 15 s long with 1 kHz sampling frequency. The method used then is much better.

Chmelka et al. [65] implemented a WF on the data from the common standards for electrocardiography multilead atlas library with 500 Hz sampling frequency and with small Q and high R waves. The data chosen by them also contained remarkable changes in signal in the QRS complex. With descending noise level, this filter stopped working.

Sun et al. [66] implemented a morphological filtering algorithm using modified morphological operators for baseline correction and noise elimination. They used the Massachusetts Institute of Technology and Beth Israel Hospital (MIT-BIH) arrhythmia database [67] and came to the conclusion that selection of the structuring element sequence depends on the pulse rate and the shape of the pulse signal. This algorithm is suitable for the conditioning the fECG signal as preprocessing.

### 2.6. De-Shape Short Time Fourier Transform and Nonlocal Median

Short time Fourier transform was created to modify limitation of discrete Fourier transform. This method provides a good compromise between the time and frequency representations of the input signal, but STFT is not very accurate for signal analysis of non-stationary and time varying noise. A new method for fECG extraction is composed of de-shape STFT in order to accurately obtain the maternal and fetal R-peaks and estimation of fHR and mHR, and NM, for estimation of maternal and fECG waveforms. It comprises a few steps. Preprocessing, running de-shape STFT to estimate the maternal instantaneous heart rate, obtaining maternal R-peaks by beat tracking and dynamic programming, estimating the mECG morphology by the nonlocal median, getting the fHR and obtaining the fECG signal [68].

Su and He [68] developed this algorithm of fECG extraction and evaluated it on synthetic and real data. They used a simulated fECG signal database (fecgsyndb) [69], which is a publicly available simulator that generates simultaneously the mECG and fECG signals in 34 channels, which is 5 min long and with sampling rate 250 Hz, and real data from adfecgdb [10,47,48,49,50], which contains five records from different women that are 5 min long with a sampling frequency of 1 kHz and with 16-bit resolution, and the ECG physionet challenge 2013 database [50], which consists of 175 four-channel abdominal fECG recordings with the durations of 1 min, 10 min, and 60 min, sampling frequency 1 kHz and 12-bit resolution. They came to the conclusion that this method extracts fECG very well and can utilize hidden information inside the aECG signal like both the frequency and energy information and the nonlinear relationship between consecutive cardiac activities.

### 2.7. Single Channel Blind Source Separation

Single channel BSS is based on using multi-algorithm fusion to process a single abdominal signal. This method divides signal into intrinsic mode functions (IMFs) by using EMD, maps single channel into multiple channels. It uses the bootstrap method, the Hough method, the Akaike information criterion method and principal component analysis (PCA) to estimate the independent component number. Then, the four numbers obtained are fused utilizing particle swarm optimization (PSO) to determine the accurate number [70].

He et al. [70] developed the SCBSS method. For the evaluation, they used a single channel signal mixed with four man-made signals and the MIT-BIH arrhythmia database [67], where two ECG signals were chosen. They concluded that this method could accurately determine the number of babies during pregnancy.

### 2.8. Template Subtraction

This method uses the repeatability of the maternal component for obtaining the fetal component and can be applied to a single-lead abdominal recording or multi-lead abdominal recordings. Every maternal beat is reconstructed by a common beat waveform, which is the template. This template is determined directly from the abdominal signal and contains a number of signal processing steps. In the first step, maternal R-peaks are obtained and the whole maternal beat is segmented to have the corresponding PQRST complexes. Then, averaging is conducted in order to obtain the template. The template can be computed from each segmented beat by using particular adaptive filters. Finally, all maternal beats are reconstructed and concatenated to estimate the maternal component, which is subtracted from the abdominal signal [45].

Agostinelli et al. [71] introduced a template-based method called segmented-beat modulation, which works under the hypothesis of knowing R-peaks, to estimate fECG signal of almost the same quality as direct fECG signal. For evaluation, they used real data from adfecgdb [10,47,48,49,50], which contains five records from different women that are 5 min long with a sampling frequency of 1 kHz and with 16-bit resolution. As evaluation parameter, they used SNR to quantify quality of template-based method. They conclude that template-based method achieved fECG signals correlated to direct fECG signals. Moreover, they claimed that this method strongly improves the accuracy of fECG estimation and may contribute to the spread of this non-invasive technique into the clinical practice.

### 2.9. Sequential Total Variation Denoising

This method is based on total variation denoising (TVD), which is widely used to reduce noise in image processing, and the TS based method called TS${}_{\mathrm{PCA}}$; this method was used since it showed better performance than other TS methods. Firstly, TVD is applied for a filtering aECG signal. Then, the TS method is used for mECG extraction and, finally, TVD is applied again, as a cascaded process, to the residual signal for estimating an fECG signal [72].

Lee et al. [72] developed the STVD method and, for the evaluation, they used database fecgsyndb [69], which is publicly available. The simulator generates simultaneously the mECG and fECG signals in 34 channels, which are 5 min long and with a sampling rate of 250 Hz, and the real data from the ECG physionet challenge 2013 database [50], which comprises 175 four-channel abdominal fECG recordings with the duration of 1 min, 10 min, and 60 min, a sampling frequency of 1 kHz and the 12-bit resolution. As preprocessing, they used the Butterworth band-pass filter with 3 Hz and 90 Hz cutoff frequencies and a notch filter. They compared STVD with the extended KF framework, TS${}_{\mathrm{PCA}}$ and combined TVD with TS${}_{\mathrm{PCA}}$. They concluded that STVD is able to effectively detect true fECG R-peaks and to decrease the error rate of detection and can be used for monitoring the fHR because this method had a low computational load.

### 2.10. Empirical Mode Decomposition

Empirical mode decomposition is an automatic method and a fully data adaptive method which decomposes non-stationary and nonlinear signals into oscillating components with some basic properties. This method decomposes time series into a sum of bandlimited functions by empirically identifying physical time scales intrinsic to the data. All bandlimited functions called IMFs contain two conditions. First, the number of extrema and the number of zero crossings must be equal or differ at most by one in the whole record. Second, in the whole record, the mean value of the envelope defined by the local maxima and the envelope defined by the local minima is zero. It is necessary to observe that the instantaneous frequency will not have redundant fluctuations as induced by asymmetric waveforms. One condition is practically the same as the narrow-band requirement for a stationary Gaussian process and another is the local requirement induced from the global one. Empirical mode decomposition depends on the number of frequency components and the amplitude of each component of the processed signal and starts extraction of high frequency components and iterates its way through to the lower frequency components. This method has six basic steps. The first step comprises identification of the maxima and minima of the time series. The second step is generation of the upper and lower envelopes by connecting the maxima and minima separately with the cubic spline interpolation. The third step is determination of the local mean. The fourth step is subtraction of IMF from the original signal for creating the zero local mean. The fifth step is checking whether the output created function of the zero local mean is IMF or not based on the conditions described [5,73].

### 2.11. Summary of Single Channel Methods

Table 2 shows a summary of different single channel methods. An objective comparison of the currently used methods is a challenging task since the authors differ in the dataset, evaluation methods, and so on. Therefore, to assign the techniques to a specific class (e.g., performance, SNR improvement, computational cost, real-time and implementation complexity), we introduce following subjective criteria, which, in some way, reflect a fuzzy logic approach:

- Overall performance—this parameter reflects the robustness of the method used, and it can be divided into three groups:
  - Low—methods suitable primarily for NI-fECG preprocessing, these methods are not able to extract fECG, but only remove some specific types of interference, e.g., baseline wandering, power line interference, and so on (improvement ≤5 dB; based on fECG extraction from synthetic records).
  - Medium—methods suitable for advanced preprocessing eliminating most of the interference in NI-fECG (e.g., power-line interference, myopotentials, and electromyographic interference, isoelectric line fluctuations, motion artifacts, etc.). These methods partly suppress the maternal component allowing detection of the fQRS complex and thus fHR determination; further morphological analysis is not possible (improvement ≤20 dB; based on fECG extraction from synthetic records).
  - High—the most powerful comprehensive NI-fECG processing methods that provide information on fHR, and fECG morphology—PR, QT, ST intervals and so on (improvement ≥20 dB; based on fECG extraction from synthetic records).
- SNR improvement—this parameter takes into account the improvement of SNR, can be divided into three categories: low, medium, and high. It should be noted that the SNR parameter objectively determines the efficacy of the method with regards to the reference; however, in terms of the clinical use, the used SNR as a parameter may be very misleading. The methods that show excellent SNR improvement can be very inaccurate in fQRS complex detection.
- Computational cost—this parameter evaluates the demands of the methods in terms of computational complexity; the categories are low, medium, and high.
- Real-time—parameter defining whether the method can be used in online mode (real-time) from the point of view of its feasibility using currently available hardware devices in clinical practice.
- Implementation complexity—this parameter, divided into three categories low, medium, and high, evaluates the overall complexity in terms of its deployment in clinical practice. The complexity of hardware and software must be economically viable for the public health system to be available to all pregnant women.

## 3. Multichannel Signal Sources

This group of methods includes mainly methods based on BSS, which is very promising and developing work in biomedical signal processing and not only for fECG extraction. Fetal ECG is obtained by means of estimation of independent sources for fetal cardiac bioelectric activity [2]. These methods are used to extract unobserved signals (sources). The sources are assumed to be statistically independent of a known mixture of these signals [57]. Blind source separation is divided into methods based on higher-order statistical (HOS) information, which is performed by independent component analysis (ICA), and methods based on second-order statistics (SOS), which is performed by singular value decomposition (SVD), PCA or period component analysis (πCA). However, there are many other methods, which are not based on BSS, such as sequential analysis (SA), Barros’s algorithm (BA), Zhang’s algorithm (ZA), extraction method using its skewness value which lies in a specific range (in this article referred to as skewness method, SM), quality index optimization (QIO), polynomial matrix eigenvalue decomposition (PEVD), fuzzy C-means clustering method (FCM), compressed sensed (CS), maternal component suppression method (MCSM), πTucker method, multivariate empirical mode decomposition (MEMD), TS, etc. Table 3, at the end of Section 3, shows comparison of the different multichannel methods.

### 3.1. Independent Component Analysis

Independent component analysis is the most widely published and used non-adaptive method of extraction of fECG signal. This method assumes that components are statistically independent and require as many electrodes placed on the maternal abdomen as the number of uncorrelated signal sources, so, in case of extraction of a fetal and maternal component from abdominal signal, ICA needs minimum two electrodes. It is not useful to use too many electrodes because each electrode carries its own noise. In preprocessing of ICA, centering is applied, which makes the vector a zero-mean variable, along with whitening, which creates a new vector whose components are uncorrelated and their variances equal unity. Many ICA-based techniques have been proposed, e.g., fast ICA algorithm (FastICA), single channel ICA (SCICA), joint approximate diagonalization of eigen matrices algorithm (JADE), minimum Renyi’s mutual information algorithm (MeRMaId), InfoMax algorithm, multidimensional ICA algorithm (MICA), nonparametric ICA algorithm (NpICA), flexible ICA algorithm (FlexiICA), orthogonal-group ICA neural algorithm (OgICA), adaptive ICA algorithm based on fully-multiplicative orthogonal-group (FastAdaptiveOgICA), Pearson ICA, etc. In extraction of fetal R-peaks, which overlaps with mQRS complex, ICA has accuracy nearly to 97.47% [57].

Ahuja et al. [74] showed a function of the FastICA algorithm. The problem of ICA is to obtain matrix X and A, so that the column vectors of matrix $X^{T}$ were independent. Kurtosis is a maximum for column vectors and for independent signals compared to mixed signals, so independent signals are also more non-Gaussian compared with mixed signals. The algorithm of FastICA starts with converting a mixed signal $y_{1}\left(t\right)$ and $y_{2}\left(t\right)$ into signals $z_{1}\left(t\right)$ and $z_{2}\left(t\right)$, so, when a covariance matrix is computed using the converted signals, $z_{1}\left(t\right)$ and $z_{2}\left(t\right)$ form an identity matrix. In the next step, values for matrix B, such that $B^{T}B=I$, are initialized and, then, update elements of matrix B by using iteration formula are initialized too. Other elements of matrix B are computed using the same procedure. The columns of matrix B are made orthogonal to each other along with repeating the updates of all elements and making columns orthogonal for *N* iterations. Finally, the independent signals are given by multiplying $B^{T}$ with the Z matrix. For the evaluation of FastICA, they used eight lead electrode signals and came to the conclusions that FastICA is clinically important for estimating the different health related parameters, the regarding fetal ones. They concluded that FastICA algorithm is very efficient and fast for extraction of independent components.

Pani et al. [75] compared JADE algorithm, online JADE algorithm (OL-JADE) and block-by-block application of JADE (BB-JADE) over short-length segments with manual reordering of the sources. This ICA-based method called JADE consists of the SOS stage, which provides centering and whitening, and HOS stage. Whitening decorrelates and orthogonalizes the original mixtures and, due to this fact, it reduces the number of parameters to estimate, so only rotation (provided by the HOS) is required to identify the independent sources. It is a batch algorithm, which is focused on a segment of the data of interest containing enough statistical information on the independent components, but JADE is highly sensitive to noise. For the evaluation, they used data from a publicly available database [76], consisting of eight real recordings (five abdominal, three thoracic) with 12-bit resolution, 10 s long and with a sampling rate of 250 Hz, and from a database by the courtesy of prof. L. De Lathauwer [77] consisting of eight real recording (five abdominal, three thoracic) with 12-bit resolution, 1 min long and with a sampling rate of 500 Hz. They resampled the second database at 250 Hz because it was found out that the higher sampling rate is not useful for better separation. For the evaluation, they used root-mean-square errors between the homologous fECG estimated sources within T-wide segments. They evaluated also the separation results and the robustness to permutations. They came to the conclusion that JADE algorithm has, due to a not-perfect online sample-by-sample SOS stage, bad robustness to noise and contains permutations on the noise channels, but all the useful signal sources do not seem to be affected by permutations. The proposed solution is very promising and still improving.

Ananthanag et al. [78] introduced and compared ICA-based algorithms, such as JADE algorithm, fixed-point algorithm, InfoMax algorithm and Comon’s algorithm. Kurtosis and computations, which can be performed either in a hatch mode or in a semi-adaptive manner, use fixed-point algorithm. For updating the separation matrix and for finding the independent components, a one at a time fixed-point algorithm uses the deflation approach. Subsequently, the fixed-point algorithm started to use a hyperbolic tangent, exponential or cubic functions as a contrast function. Another ICA-based unsupervised learning algorithm called InfoMax, developed by Bell and Sejnowski, is based on entropy maximization in a single layer feed-forward neural network. This method is using the idea that, by maximizing the joint entropy of outputs of a neural processor, the mutual information among the output components can be minimized. The algorithm developed by Comon is based on the contrast function based on minimization of mutual information between the components at the output of the separator. The contrast function is directly related to Kullback–Leibler divergence between the output vector probability density function (pdf) and its pdf if it was made of independent components. Evaluation of accuracy of these methods was made on simulated data, which was created by taking two different ECG signals (fECG and mECG) and adding random white Gaussian noise. They came to the conclusion that all the BSS algorithms can extract fECG with very good accuracy, which is very useful especially in multi fetal cases. They achieved more accuracy when they used a more observed signal or electrodes. If SNR was worse, then P and T waves were lost in the noise, but still these ICA-based algorithms were able to detect the R wave.

Marossero et al. [79] used a MeRMaId algorithm to compare FastICA and InfoMax. Algorithm MeRMaId works in four steps. The first one is initialization of the given angles to all zeros or randomly. Then, the whitening matrix in offline separation or by updating adaptive PCA algorithm online is computed. Then, if offline separation is used, use the batch gradient or, if online separation is used, use the stochastic gradient, which uses only certain number of samples, including the most recent sample at certain time. In case the stochastic gradient is used, in the last step, the given rotation angles are updated using the steepest descent. The methods were compared using three parameters, such as algorithmic complexity, robustness and signal separation performance, which is compared by signal-to-distortion ratio (SDR). For the evaluation, they used an artificial mixture of two clean ECG signals with an original sampling frequency of 500 Hz and 5 min long real data, which include an 8-channel mixture to extract eight source estimates with a sampling rate of 200 Hz. Preprocessing of the real data consists of order-6 FIR HPF and pre-whitening. They concluded that MeRMaId outperforms FastICA and InfoMax algorithms in both types of data. This method is shown to be more data efficient, both in batch and online operation modes, so this method can be used in real-time monitoring.

Camargo-Olivares et al. [80] came with another ICA-based technique, which is an extension of ICA and its name is MICA. This method does not assume that the components are statistically independent, but that the components can be divided into groups which are statistically independent. In this article, they proposed augmenting the number of inputs to MICA with mECG signals, which are recorded from the maternal abdomen and cleaned of fetal contributions to prevent falling of MICA. This method is unable to produce useful results, without mECG estimation before forming MICA algorithm. The block scheme contains preprocessing, mECG estimation, which is conducted by PCA method or another approach, ICA block and postprocessing. For preprocessing, they used FIR band-pass filter with cut-off frequencies at 1 and 90 Hz and a notch filter. They used one record from NI-fECG database [54,55], which were 10 s long and with a sampling rate of 1 kHz with 16-bit resolution. In ICA block, they used a first JADE algorithm, and then they tried to use another ICA-based technique, such as FastICA. They concluded that MICA is much more effective when it consists in using mECG signals as inputs to MICA extracted from abdominal signals.

Sevim et al. [81] used a kernel-based model for directly estimated pdfs from data by using the kernel density estimation technique for building another ICA-based technique called NpICA. This method at the same time estimates unknown pdfs of the source signals and the linear operator, which allows the separation of the mixed signals. In the experimental part, they used synthetic datasets linearly mixed by 1000 randomly generated mixing matrices with signal lengths ranging between 512 and 4608 and with additional noise source by using 1000 Gaussian random signals. The evaluation of the synthetic data was perform by using median SNR and compared with FastICA and JADE algorithms. In the next experiment, they used real data only on NpICA algorithm and this data was obtained from nine different skin electrodes located on different points of the maternal abdomen. They came to the conclusion that NpICA outperformed other known ICA algorithms, such as FastICA and JADE. This method is very robust and showed very good performance, especially in recordings with a high signal length, but this superior performance was attained at the expense of increased computational complexity.

Ye et al. [82] proposed a fast and adaptive ICA-based algorithm called FastAdaptiveOgICA, which is based on a fully-multiplicative orthogonal-group, which can instantaneously separate mixtures of sub-Gaussian source signals, super-Gaussian source signals and also can separate skewed or near Gaussian signals. For the comparison, they used an OgICA algorithm, which is fully-multiplicative, a batch learning algorithm for neural independent component analysis, InfoMax algorithm and FlexiICA algorithm, which can separate mixtures of sub-Gaussian and super-Gaussian source signals with self-adaptive nonlinearities. The data was obtained from benchmark data file from ICALAB toolbox [83] and a database developed by De-Moor [53], which were 10 s long and with a 250 Hz sampling rate. They concluded that FastAdaptiveOgICA was successfully applied to obtain fECG signals with better separation performance and faster convergence speed than other ICA-based algorithms that were compared.

### 3.2. Singular Value Decomposition

It is a spatial filtering technique and a decomposition method that are driven by the data that creates the required basis functions from the data itself, by maximizing several statistical quantities of signal segregation [2]. This method is based on a matrix transformation of one vector space into another and the basic mathematical equation is $Y=U^{T}X$. The algorithm of this method is very computationally demanding [84].

De Lathauwer et al. [85] compared the ordinary SVD technique, quotient SVD technique and multilinear SVD technique. For the experiment, they used 8-channel 1 min long signals with sampling a frequency of 500 Hz and evaluated by computational complexity, robustness, required interaction with the user amount of provided information. They concluded that the mixing matrix in multilinear SVD can be estimated in an unsupervised way, but this technique is computationally more complex. They marked decomposition methods as currently the most common and effective methods of fECG extraction.

### 3.3. Principal Component Analysis

This method reduces the number of dimensions from a numerical measurement of several variables. During the search for simplifying a statistical problem, PCA loses minimal information. This method can be also used for searching for linear combination for separation of signals from sources which are statistically independent. This is done by determining the data with a new coordinate system and this operation is bidirectional and no information is lost [86].

Bacharakis et al. [87] compared the PCA method with high-order SVD and high-order eigenvalue decomposition (EVD). They used eight real records from pregnant women, which are 10 s long and with a sampling frequency of 500 Hz, and they came to the conclusion that the PCA method worked worse than other methods compared during fECG extraction.

### 3.4. Period Component Analysis

Periodic component analysis uses destructive interference for denoising and constructive interference for improving the periodic components of the frequency spectrum. It removes inefficacy of autocorrelation at the pitch period, so πCA does not need log delay lines and correlates signals at a clock rate on the order of the actual pitch to be compared with the original sampling rate. This method seeks for the linear mixture with a maximal periodic structure that minimizes the measure of periodicity [88].

Kharabian et al. [88] used πCA for fECG estimation and then Hilbert transform to enhance R-peaks. For the evaluation, they used eight synthetic records with a sampling frequency of 500 Hz and with added noise through the generator. They compared this method with JADE algorithm and concluded that measures of periodicity are an opportunity for using a priori information to have better extraction of the required statistics and that ranking the components is a helpful feature for automating mECG filtration.

### 3.5. Sequential Analysis

Sequential analysis uses a priori information about the interference for the extraction of fECG signal. This method consists of a baseline wander remover, a power-line interference canceller, a QRS detector, an mECG canceller and an fECG detector [89].

Martens et al. [89] introduced a new method called SA for fECG extraction. They compared SA with JADE algorithm on 20 measurements recorded with 20 pregnant women from the 18th to 38th week of pregnancy. They concluded that SA outperforms ICA with a fHR detection rate of 85%, compared to 60% of ICA, so SA is more robust than ICA. This method is great, especially in recordings with low SNR.

### 3.6. Barros’s Algorithm

Barros’s algorithm belongs in blind source extraction (BSE) algorithms, which are learning algorithms that can extract a single source signal from a linear mixture of source signals unlike BSS methods, which extract all the source signals. It is a very simple batch learning algorithm for semi-blind extraction of a specific signal with a temporal structure. The signal of fECG is extracted from linear mixtures. Although this method uses a concept of sequential blind extraction of sources and ICA, this method does not assume that the sources are statistically independent and do not perform the extraction blindly, but semi-blindly. It uses a priori information about the autocorrelation function of the primary sources for fECG extraction. The performance of this algorithm depends on the precise estimation of the period of the fetal component [90].

Barros and Cichocki [90] used this method for fECG extraction. They used a database developed by De-Moor [53] containing recordings that were 10 s long and with a 250 Hz sampling rate, and came to the conclusion that this method is able to work efficiently for fECG extraction, but this method extracts signals as long as they are decorrelated and shows a temporal structure. This method also had a high speed of convergence when only a few iterations were needed to achieve convergence.

Zhang and Ye [91] developed extended BA, which can extract completely unknown sources that have autocorrelation properties, unlike BA, which is limited by requiring a priori knowledge about the sources’ autocorrelation information and its use on unknown sources for extraction of fECG. Their proposed method applies BSS technique to the autocorrelation functions of the sensor signals and, for optimal time delay, it obtains, in preprocessing, regularly reappearing peaks in the autocorrelation function. They also used, for the evaluation, the database developed by De-Moor [53] containing recordings that were 10 s long and with a 250 Hz sampling rate. They came to the conclusion that this extended version can extract fECG and, in general, the unknown sources as long as they have autocorrelation properties.

### 3.7. Zhang’s Algorithm

This method extracts fECG signal from aECG signals by using a priori knowledge about the range in which its kurtosis value lies. The algorithm is based on evaluation of a range of the kurtosis of fECG signal. The main problem is that the estimation error of the kurtosis very much affects the performance of this method [92].

Zhang and Yi [92] developed an extraction method using its kurtosis value which lies in a specific range. They used synthetic data which contains four independently 100 times mixed artificial source signals with zero means, unit variances and 3000 samples, and real data from the database developed by De-Moor [53] containing recordings that were 10 s long and with a 250 Hz sampling rate. They came to the conclusion that this method is useful to extract fECG signal whose kurtosis value lies in a specific range.

### 3.8. Skewness Method

It is a lightweight algorithm that uses pre-knowledge about its skewness for the extraction of fECG signal. By skewness, a cost function, which updates the weight vector for the extraction of fECG signal, is defined. This method uses a range for fetus’s skewness value, which was found by comprehensive experiments on real and synthetic world data [93].

Jafari et al. [93] used this method for fECG extraction and evaluation on SNR and computer execution time. They used the database developed by De-Moor [53] containing recordings that were 10 s long and with a 250 Hz sampling rate and came to the conclusion that this method improved the quality of the extracted signal by increasing, and the computational cost required for extracting fECG was decreased.

### 3.9. Quality Index Optimization

Quality index optimization is a new approach to fECG extraction from aECG signals. This method uses the characteristics of pseudo-periodicity and the time shape of QRS and consists of devising a quality index (QI) that synthesizes the characteristics of pseudo-periodicity. For fQRS extraction, one QI for fECG (fQI) and one for mECG (mQI) are created. This method comprises a few steps, such as signal pre-processing, mQRS-enhanced signal extraction, which is found by the linear combination that maximizes the mQI, mQRS detection, mECG component approximation and cancelling by weighted SVD, fQRS-enhanced signal extraction, which is found by the linear combination that maximizes the mQI, and fQRS detection [94].

Varanini et al. [94] compared the QIO method with some ICA-based methods. They used 75 records with a sampling frequency of 1 kHz from the ECG physionet challenge 2013 database [50] and concluded that QIO outperforms the ICA-based methods in fQRS detection. This method eliminated the problem of ICA, which automatically selects fECG or mECG among the estimated independent sources and can be used in the presence of a weak fECG signal.

### 3.10. Polynomial Matrix Eigenvalue Decomposition

Polynomial matrix eigenvalue decomposition is used for estimating the broadband noise subspace. This method uses the second-order sequential best rotation algorithm, which is an iterative, time-domain algorithm based on the SOS and gives improved strong decorrelation and spectral majorization. Based on the decomposition, this method allows estimation of the subspace of fECG signal. This method is a blind technique that does not have prior knowledge of the sources or the mixing matrix, but only necessitates the knowledge of the space-time covariance matrix. The power of mECG is sufficiently compared to fECG in the recordings so as to separate from aECG [95].

Redif [95] introduced a PEVD method and tested it on synthetic data from the MIT-BIH arrhythmia database [67], which consists of three half-hour recordings with a sampling frequency of 360 Hz, and the ECG physionet challenge 2013 database [50], which consists of 175 four-channel abdominal fECG recordings with the durations of 1 min, 10 min, and 60 min, a sampling frequency of 1 kHz and 12-bit resolution. They compared this method with SVD and ICA-based methods and came to the conclusion that PEVD is able to estimate the fetal R waves with good accuracy. They concluded that PEVD improved accuracy and robustness. This method does not need mECG as a reference signal and the experiments indicate that its sensitivity to sensor placement is low.

### 3.11. Fuzzy C-Means Clustering Method

The fuzzy C-means clustering method proposes a simple and effective method to judge the case of multiple births and estimate the number of multiple births. This method provides the prior knowledge for the subsequent blind extraction method of fECG. The goal of this method is selecting *c* initial values as the initial clustering centers and then separating data objects into clusters through iteration, so then the same clusters are minimized, and the different ones are maximized [96].

Tan et al. [96] used an FCM method for extraction of fECG. They used two aECG signals from pregnant women, which are 7 s long and with a sampling frequency of 500 Hz. They marked FCM as extremely effective, accurate and safe in fECG extraction and simple for judging the case of multiple births.

### 3.12. Compressed Sensed

This method is based on the sparse representation of the components, which are obtained from ICA, applied in the compressed domain. It is a sensing and sampling technique that allows for recovering sparse signals from fewer samples than can be done by the Shannon sampling theorem. It is assumed that a small collection of linear projections contains enough information for the reconstruction of sparse signals. Compressed sensed contains acquisition and compression stages and it is a really interesting method for wireless bio-signal monitoring systems [97].

Da Poian et al. [97] developed CS method and compared with FastICA algorithm. They used database adfecgdb [10,47,48,49,50], which contains five records from different women that are 5 min long with a sampling frequency of 1 kHz and with 16-bit resolution, and the ECG physionet challenge 2013 database [50], which consists of 175 four-channel abdominal fECG recordings with the durations of 1 min, 10 min, and 60 min, a sampling frequency of 1 Hz and 12-bit resolution. They concluded that this method can be applied for compression of abdominal fECG and for obtaining real-time information of the fHR.

### 3.13. Maternal Component Suppression Method

This method relies on determination of the maternal PQRST complex and the following subtraction during the consecutive maternal cardiac cycles, which is done by synchronizing the template PQRST and the successive complex in time. The maternal component suppression method first determines the fiducial points $t_{i}$, then determines the PQRST complex by averaging the adequate complexes, then determines the $a_{i}$ factors and, at the end, subtraction of the template from abdominal signal in the fiducial points [98] is performed.

Jezewski et al. [98] used MCSM and compared it with three other selected methods for mECG suppression using dedicated coefficients. They used three records, which contained four abdominal signals. They came to the conclusion that this method does not require any thoracic signals and does not require a strictly determined location of measurement electrodes. They also concluded that this method allows complete suppression of mECG signal without influence on fECG signal, but this method does not suppress all other noise components.

### 3.14. πTucker

This method uses cardiac signal morphology, which is its quasi-periodic nature, and a penalized objective function for Tucker decomposition for extraction of fECG signal from aECG signals. It is an iterative algorithm, which can automatically select the desired components in the source space [99].

Akbari et al. [99] used πTucker method for fECG extraction and compared it with ICA-based methods. For the evaluation, they used synthetic data and real data from database adfecgdb [10,47,48,49,50], which contains five records from different women that are 5 min long with a sampling frequency of 1 kHz and with 16-bit resolution. The results showed good performance of this. This method needs only 20 iterations for a satisfactory error in the extracted fECG.

### 3.15. Multivariate Empirical Mode Decomposition

This method is based on EMD, which is a fully data-driven method for nonlinear and non-stationary real-world signals. It divides the signal into a finite set of IMFs. The first step of this method comprises elimination of the noisier noisy channels based on comparison of similar indexed IMFs, which were found by MEMD. Then, denoising of the remaining noisy channels is performed by eliminating the similarly indexed IMFs. Finally, an mECG signal is eliminated from aECG signals and fECG signal is detected by CWT [100].

Gupta et al. [100] used the MEMD method on real data for extraction of fECG. They used data from the database adfecgdb [10,47,48,49,50], which contains five records from different women that are 5 min long with a sampling frequency of 1 kHz and with 16-bit resolution, and from another database [101], which contains one record from a mother in gestational age of 40 weeks and the sampling rate of this record was 1 kHz. They came to the conclusion that MEMD had high value of the cross correlation between the detected and true fHR signals, so this method can be used for fHR monitoring.

### 3.16. Summary of Multichannel Methods

Table 3 compares different multichannel methods. For the relevant comparison of all the introduced methods, the same assessment criteria as in Table 2 was used. It can be stated that most multichannel methods achieve better results than single-channel methods.

## 4. Hybrid Methods

A large number of studies address the possibility of combining either adaptive methods with one another, non-adaptive methods with one another, or combinations of adaptive and non-adaptive methods together in order to create hybrid methods. Very often, non-adaptive methods, such as preprocessing, are used for adaptive algorithms. The non-adaptive methods partially separate the components and make extraction easier for the adaptive system. This section is dedicated to these hybrid methods, which improve accuracy compared to the use of only one method. Table 4, at the end of Section 4, shows the comparison of the different multichannel methods.

### 4.1. ICA-EEMD-WS

This adaptive integrated algorithm for fECG extraction is called ICA-EEMD-WS because it consists of ICA, ensemble EMD (EEMD) and wavelet shrinkage (WS). First, the FastICA algorithm is applied to separate the mixed aECG signal and to obtain the noisy fECG. Then, the noisy fECG is cleared by partial reconstruction from IMFs, which means that EEMD decomposes the noisy fECG by a three-step integrated algorithm. Finally, WS is used to reduce the high-frequency noise. The baseline wander and other noises are eliminated directly. Finally, fECG is reconstructed by the denoised IMFs in group one and those reserved IMFs in group two [102].

Liu and Luan [102] developed ICA-EEMD-WS and compared it with Butterworth filter, pure WS and EMD-WS. For the evaluation, they used synthetic data from a realistic NI-fECG generator that uses the Gaussian ECG model [103], which consisted of six multichannel signals with 1200 samples and a sampling rate of 1 kHz, and 500 single channel signals with different noises. They also used the MIT-BIH arrhythmia database [67], which consists of three half-hour recordings with a sampling frequency of 360 Hz, and data from database adfecgdb [10,47,48,49,50], which contains five records from different women that are 5 min long with a sampling frequency of 1 kHz and with 16-bit resolution. They concluded that this method obtains a larger SNR and correlation coefficient, and also a smaller mean square error compared to other tested algorithms and, basically, outperforms the conventional algorithms. This method could be used in real-time monitoring of fHR and mHR if EEMD algorithm would be faster.

### 4.2. ICA and AF

Gupta et al. [104] introduced a hybrid method, which consists of an adaptive filter (AF) and non-adaptive algorithm. A four-channel adaptive mECG canceller (FAMC) followed by an adaptive fECG enhancer (AFE) is an approach called FAMC-AFE. The adaptive canceller FAMC uses three orthogonal thoracic signals and one abdominal signal to estimate mECG signal and to extract the fECG signal. This hybrid method combines the above-mentioned adaptive approach FAMC-AFE and ICA algorithm called BSS-ICA AFE. They evaluate the function of this method on synthetic data from the MIT-BIH arrhythmia database [67], which consists of three half-hour recordings with a sampling frequency of 360 Hz, and on real data, from database developed by De-Moor [53] containing recordings that were 10 s long and with a 250 Hz sampling rate. They came to the conclusion that this hybrid method outperforms ICA, the scheme FAMC-AFE and provides superior results compared to the old schemes.

### 4.3. ICA and PF

Kotas [105] used a combination of multichannel ICA and single channel PF of the time-aligned beats for fECG extraction. Projective filtering can be applied to enhance the partially separated mECG and then mECG can be reconstructed and subtracted from aECG for obtaining fECG. By the second application of ICA, the signal can still be enhanced. A block diagram of this combination consists of linear filters for preprocessing, ICA for source signals estimation, selection of the maternal source signals, PF of the maternal source signals, reconstruction of mECG contained in the respective channels and the second application of ICA for fECG enhancement. In this method, he used an ICA-based method called a JADE algorithm. For the evaluation, he used signals of four patients, which were recorded with the use of the four abdominal leads [106]. He concluded that this combination allows to extract the fECG effectively in cases when ICA alone fails or gives poor results and that this combination can be a useful device for prenatal diagnosis.

### 4.4. π-ICA

Sameni et al. [107] introduced π-ICA algorithm partially based on πCA and generalized EVD. This method tries to find any periodic structure, which is synchronous with the reference ECG R-peaks estimated from the clean ECG reference. The experiment was conducted on real data from database developed by De-Moor [53] containing recordings that were 10 s long and with a 250 Hz sampling rate. They discussed several benefits of this method over conventional source separation techniques, e.g., that this method replaces the independence criterion of ICA with a periodic temporal structure criterion, which is interesting for the cardiologists because its periodic structures are repeated in each ECG beat. This method replaces the iterative ICA algorithm with a closed-form solution consisting of an initial R wave detection step, so this method is faster. This method provides a benefit consisting of the fact that π-ICA ranked the extracted components according to their degree of synchronization, their periodicity, with the R-peaks. However, the solutions of generalized EVD problems are generally more susceptible to noise, but the results are still robust to deviations of the heartbeat and noise.

### 4.5. ICA and PCA

Martín-Clemente et al. [108] used a combination method which consists of a dimensionality reduction step and a computationally light postprocessing stage used to enhance the fECG signal. The dimensionality reduction step is based on PCA and reduces the number of signals under consideration; it is intended to speed up the estimation process and to reduce appreciably the mECG interference. The computationally light postprocessing stage is based on an ICA method, which is based on maximizing the kurtosis. Then, the PCA method is applied again for reducing the dimensionality in one unit. This algorithm is repeated until all the wanted signals are recovered. They evaluated this combination on open source ECG toolbox for generating synthetic mECG and fECG mixtures with realistic ECG noises, which consists of eight simulated recordings of 5000 samples, and on the database by the courtesy of Prof. L. De Lathauwer [77] consisting of eight real recording (five abdominal, three thoracic) with 12-bit resolution, 1 min long and with a sampling rate of 500 Hz. They compared this combination with FastICA, JADE, π-ICA and Pearson ICA. They concluded that this combination is simple, fast and can be used for designing battery-powered devices for monitoring fHR.

### 4.6. ICA and SVD

Gao et al. [109] used a combination of SVD of the spectrogram and an iterative application of FastICA on both the spectral and temporal representations of the ECG signals for extraction fECG from a single channel signal. The main idea of this combination is to project aECG signal into a higher dimension and to use the assumption of statistical independence between the components to estimate fECG. First, SVD helps with separability of the components and, then, FastICA contributes to the independence of the two components. They compared this combination with SVD and evaluated it by fetal heartbeats detection on a synthetic mixture, which is constructed from two simulated ECG complexes, and on a real signal 10 min long with a sampling rate of 300 Hz. They concluded that this combination is more accurate than using SVD and that this combination falsely detected the occurrence in cases where the maternal and fetal heartbeat coincide.

### 4.7. BA and ZA

Yang and Lei [110] developed a novel BSE algorithm combing BA [90], which uses periodicity, and ZA using kurtosis [92]. They compared this combination on an original method by Barros and Zhang by using real data from the database developed by De-Moor [53] containing recordings that were 10 s long and with a 250 Hz sampling rate. They concluded that this combination of periodicity and kurtosis is not sensitive for the estimated error of the period of fECG compared with BA that is not sensitive for the estimated error of the kurtosis range of fECG compared with ZA. All three experiments showed better performance of this combination.

### 4.8. SVD and PC

Ayat et al. [111] used a combination of SVD and polynomial classifiers (PC) for fECG extraction. By using SVD, mECG, which is used with aECG as an input for PC that extract fECG by exploiting the dynamics and the nonlinearities of the mECG, is estimated. This combination uses a single channel signal source, so it requires simpler hardware and makes long term recordings of the fECG possible. For the evaluation, they used a synthetic signal, which is generated from two completely different ECG signals, and real data from the database by the courtesy of prof. L. De Lathauwer [77] consisting of eight real recordings (five abdominal, three thoracic) with 12-bit resolution, 1 min long and with a sampling rate of 500 Hz, and a dataset recorded in AI-Wasl Hospital in Dubai. They came to the conclusion that this combination is very effective and capable of extracting fECG from a single lead.

### 4.9. PN and SGSF

Ayat et al. [112] developed a two-tier technique for fECG extraction from a single abdominal record. First, a SGSF is applied for mECG estimation from aECG signal by suppressing the fECG component which can be observed as noise of the overlapping frequency contents with the wanted mECG signal, and, then, this mECG is nonlinearly aligned with the abdominal signal using polynomial networks (PN) to extract the fECG. They compared this new technique with SVD method and used synthetic data, which is created by one set of real normal resampled data and added to the other real normal ECG signals, and real data from the ECG physionet challenge 2013 database [50] and from a dataset recorded in AI-Wasl Hospital in Dubai. They came to the conclusion that this new combination of SGSF and PN provides better results than SVD method and removes the requirement for PN that use one abdominal and one thoracic signal.

### 4.10. Summary of Hybrid Methods

Table 4 compares different hybrid methods. For relevant comparison of all the investigated methods, we used identical assessment in previous cases (Table 2 and Table 3). Generally, hybrid fECG processing methods outperform the rest of the introduced non-adaptive methods.

## 5. Discussion

The fetal ECG reflects the electrical activity of the fetal heart and carries a huge amount of information. Unfortunately, current technologies are able to sense and identify only a fraction of them. The fetal heart rate and its variability are the only parameters that can be obtained with sufficient accuracy at the moment (see [113,114]). However, the latest science and research suggests that the NI-fECG contains other, yet unexploited, clinically relevant data, see [115]. These are, for example, the morphology and the length of the individual NI-fECG elements, the dynamic behavior of the NI-fECG, etc. Today’s diagnostic devices are able to extract this features only by invasive approach [116,117] using a transvaginal fetal scalp electrode. However, this method can only be used after the rupture of the amniotic fluid. This invasive fECG monitoring approach makes it possible to apply the latest method to determine hypoxia based on fHR in combination with ST segment analysis (ST segment analysis using T/QRS), see [118]. However, due to its invasiveness, this method is associated with many disadvantages, such as the risk of introducing infection or significant fluctuations in the isoelectric line. The aim is to create a non-invasive variant of this method that does not endanger the mother or the fetus and could be used for pre-natal diagnosis.

Morphological analysis of the ECG generally involves a large number of different methods. These include QRS complex analysis (in particular its shape and duration), the R/S ratio (vectorcardiography), the PR/fHR ratio (inverse correlation between PR interval and fHR that changes in hypoxic states), P wave morphology/absence, PR interval, QT interval, and ST segment. An emphasis has been recently placed on the T/QRS ratio used in the above-mentioned ST segment analysis.

The comparison of non-adaptive methods summarized in Table 2, Table 3 and Table 4 offered an overview of the advantages and disadvantages of the introduced methods in terms of their overall performance, SNR improvement, computational cost, real-time, and implementation complexity. To assess the methods from the clinical point of view, we provide additional evaluation criteria illustrating the applicability in the fetal monitoring, more precisely for fHR determination and morphological analysis. In Table 5, the additional parameters are denoted and described as follows:

- fHR (R-R)—this evaluation parameter classifies the effectiveness of the investigated methods from in terms of the fHR determination based on the fetal R-R interval. There are four categories for the assessment:
  - Inaccurate—the methods are not sufficient to remove artifacts and noise sufficiently to enable the R–R interval detection; the NI-fECG processed by these methods cannot be used for fHR monitoring.
  - Moderately accurate—these methods sufficiently suppress most common interference and thus make RR interval detection possible. However, the noise is not completely eliminated and thus there are many false-detected and undetected significant complexes, i.e., sensitivity (Se) ≤ 80%, positive predictive value (PPV) ≤ 90%, accuracy (ACC) ≤ 80%, total probability of correct detection of beats (F1) ≤ 85%.
  - Accurate—these methods allow accurate detection of fHR, i.e., Se ≤ 85%, PPV ≤ 95%, ACC ≤ 85%, F1 ≤ 90%.
  - Very Accurate—these methods enable a very accurate determination of fHR and, in this case, it is a full replacement of the conventional CTG [31,40,119], i.e., Se ≤ 95%, PPV ≤ 95%, ACC ≤ 95%, F1 ≤ 95%.
- Morphological analysis (T/QRS; QT)—this parameter classifies the efficacy of the investigated methods from a deeper morphological analysis of fECG. The following categories were created for the evaluation:
  - Insufficient—these methods cannot estimate fECG in a sufficient quality for morphological analysis.
  - Moderately accurate—these methods enable morphological analysis; however, only in the case of some tested real data, the efficacy is significantly affected by gestational age, fetal position, SNR, and so on. Therefore, these methods could not be used for long-term monitoring of T/QRS ratio or QT interval.
  - Promising—these mostly hybrid methods have great potential to be used for fECG morphological analysis.
- Dataset—in this column, we provide the source of the data that was used in the studies. Databases with fECG recordings are an important part of this research. A major problem is their insufficient quantity that would be available to the scientific community. Without the databases, i.e., real recordings, the scientists can hardly verify the methods for extracting fECG and thus improve diagnostic quality contrary to the standard ECG of adults.
- Technical aspects—the last column includes the technical details of each study, e.g., number of electrodes, heterogeneity of the patient population, conditions and gestational ages, as well as data quality (duration (T), sampling frequency (Fs), amplitude resolution (res), gestational age (GA), number of electrodes (channels), type of records, and number of records).

For the reasons listed above, the major challenge nowadays is to enable morphological analysis from the non-invasively recorded signal. Some of the studies proved that it is possible, mainly using advanced hybrid non-adaptive methods [102,104,105,110]. It should be noted that specific technical aspects are associated with the morphological analysis, mainly the sampling frequency that should be higher than 500 Hz that is generally used for the fHR monitoring. Some of the databases available thus offer insufficient data for such tasks [53,57,67,69,76,77,93]. Additionally, the deployment of the electrodes for the morphological analysis is not yet standardized, but it is certain that it influences the results significantly. Therefore, we suggest that a new database should be created specifically for these purposes; it should include large values of electrodes deployed across the maternal abdomen, the sampling rate should be sufficiently high (2 kHZ or above), and it should, if possible, also include data from the fetal scalp electrode to be used as a gold standard for evaluation.

## 6. Conclusions

This article focuses on introducing different types of non-adaptive methods of signal processing. There are many applications where these non-adaptive methods can be utilized. They are used more often in the area of extraction fECG, but also in the area of fPCG or electroencephalography signal processing, voice recognition, image identification, etc. There is a large number of non-adaptive methods of signal processing and the choice of which one is used will depend on the type of signal we want to process and the result that we are trying to achieve. The most widely published methods of processing virtually all signal types are ICA, PCA and WT due to their efficiency, great accuracy and the speed of the algorithms.

Based on the extensive overview presented herein, we conclude that hybrid methods, such as ICA-EEMD-WS [102], ICA & AF [104], ICA & PF [105], and BA & ZA [110], seem to be the most promising non-adaptive methods for NI-fECG signal processing. The authors believe that the application of selected NI-fECG methods will lead to the development of a completely new diagnostic method using non-invasively recorded fHR data (fHR based on detection of NI-fECG R-R interval) to determine the fetal hypoxic state in combination with non-invasively obtained T/QRS ratio, i.e., enable non-invasive fECG ST segment analysis. Introducing this novel non-invasive diagnostic method into clinical practice should lead to a significant reduction in unnecessarily performed cesarean sections for suspected hypoxia.

## 7. Ethics Statement

The study protocol was approved by the Ethical Committee of the Silesian Medical University, Katowice, Poland (NN-013-345/02). Subjects read the approved consent form and gave written informed consent to participate in the study.

## Figures and Tables

**Table 1:** Comparison of monitoring techniques.

Method	Gestational Age Restriction	Technical Solution	Advantages	Disadvantages
NI-fECG [1,2,3,6,7,8]	≥20	Standard ECG electrodes placed on mother’s abdomen (number of electrodes differs)	Cheap Relatively accurate Easy to handle Comfortable (mobility) Continuous monitoring Fetal heart rate monitoring	Low signal-to-noise ratio Significant amount of overlapped undesired signals Without morphological fECG analysis * (ST segment analysis)
I-fECG [9,10,11]	Only during labor	Transvaginal scalp electrode (fetal scalp electrode)	fECG morphology analysis [42,43] Continuous monitoring Fetal heart rate monitoring Accurate	Invasive (risk of infection) Expensive Limited movement Uncomfortable Requires skilled personnel
CTG [9,31,32,33]	≥20 Possible to use during labor	One tranducer for fetal heart rate measurement and one tranducer for uterine contractions measurement	Smoothed heart rate time series Rather robust and reliable Most used method in clinical practice Relatively cheap Easy to implement	Not possible to assess beat-to-beat variability Ultrasound irradiation Not passive
fECHO [22,23,24,25]	≥18	The transducer in the probe serves as a transmitter and receiver for ultrasound signals	Provides reliable data on cardiac morphology as well as deviations and blood flow velocity deviations	Expensive Requires skilled personnel Ultrasound irradiation Not suitable for continual monitoring
fPCG [4,5,12,13,14,15,16,17,18,19,20,21]	≥20 Possible to use	Microphone sensors (or optical ** sensors) attached to the mother’s abdomen	Cheap No energy is transmitted Determination of multiple pregnancies Possibility of home monitoring (cell phone)	Not used in clinical practice Susceptible to movement artifacts
fMCG [26,27,28,29,30]	≥20	Fetal magnetic field detection by superconducting quantum interferencedevice sensors located near the maternal abdomen	Better morphological analysis due to higher signal-to-noise ratio	Expensive Requires skilled personnel Complexity of measurement No long term monitoring possible to date

* Current devices that are being used in clinical practice do not allow it. It can be assumed that advanced signal processing of the NI-fECG signal will soon enable morphological analysis [1,3]. ** Fiber optical sensors for measuring fPCG are being used today [12].

**Table 2:** Comparison of the different single channel methods.

Method	Overall Performance	SNR Improvement	Computational Cost	Real-Time	Implementation Complexity
WT	Medium	Medium	Low	Yes	Medium
CT	Low	Low	Low	Yes	Simple
ST	Low	Low	Low	Yes	Simple
AT	Low	Low	Low	Yes	Simple
FT	Low	Low	Low	Yes	Simple
STFT & NM	Medium	Medium	Medium	No	Medium
SCBSS	Medium	Medium	Medium	No	Complex
TS	Medium	Low	Low	No	Medium
STVD	Medium	Medium	Medium	No	Complex
EMD	Medium	Medium	High	No	Medium

**Table 3:** Comparison of the different multichannel methods.

Method	Overall Performance	SNR Improvement	Computational Cost	Real-Time	Implementation Complexity
ICA	Medium	Medium	Medium	No	Medium
SVD	Low	Low	Low	Yes	Simple
PCA	Low	Medium	Low	Yes	Simple
πCA	Medium	High	Low	No	Simple
SA	Medium	Medium	Medium	No	Medium
BA	Medium	Medium	Low	No	Simple
ZA	Medium	Medium	Low	No	Simple
SM	Medium	Medium	Medium	No	Simple
QIO	Medium	Medium	Medium	No	Medium
PEVD	Medium	Medium	Medium	No	Medium
FCM	Medium	Medium	Medium	Yes	Simple
CS	Medium	Medium	Medium	Yes	Medium
MCSM	Medium	Medium	Medium	No	Simple
πTucker	Medium	Medium	Low	No	Simple
MEMD	Medium	Medium	Medium	No	Medium

**Table 4:** Comparison of the different hybrid methods.

Method	Overall Performance	SNR Improvement	Computational Cost	Real-Time	Implementation Complexity
ICA-EEMD-WS	High	High	High	Yes	Complex
ICA & AF	High	Medium	High	No	Complex
ICA & PF	High	Medium	High	No	Complex
π-ICA	Medium	Medium	Medium	No	Medium
ICA & PCA	Medium	Medium	Medium	Yes	Medium
ICA & SVD	Medium	Medium	Medium	Yes	Medium
BA & ZA	High	Medium	Low	No	Simple
SVD & PC	Medium	Medium	Low	No	Medium
PN & SGSF	Medium	Medium	Medium	No	Medium

**Table 5:** Comparison of non-adaptive methods of fECG extraction from the point of view of obtaining clinical information.

Method	fHR (R-R)	Morphology Analysis (T/QRS; QT)	Dataset	Technical Aspects
WT [51,52,56,58] Section 2.1	Moderately accurate	Insufficient	De-Moor [53]; NI-fECG [54,55]; U. Nottingham [57]	3 synt. records [51]; T = 10 s; Fs = 250 Hz [53]; T = 10 s; Fs = 1 kHz; res = 16 b; GA = 21–40 weeks; 55 real records [54,55]; T = 60 s; Fs = 300 Hz; res = 12 b; data = 15 real records [57]
CT [57,59] Section 2.2	Inaccurate	Insufficient	—	Old method; Without tech. specification [59]
ST [60,61] Section 2.3	Inaccurate	Insufficient	—	Old method; Without tech. specification [60,61]
AT [62] Section 2.4	Inaccurate	Insufficient	—	Old method; Without tech. specification [62]
FT [64,65,66] Section 2.5	Inaccurate	Insufficient	MIT-BIH [67]	T = 15 s; Fs = 1 kHz; 15 real records [64] Fs = 500 Hz [65]; T = 0.5 h; Fs = 360 Hz; res = 11 b; 48 real records [67]
STFT & NM [68] Section 2.6	Accurate	Insufficient	fecgsyndb [69]; adfecgdb [10,47,48,49,50]; ECG physionet challenge 2013 [50]	T = 300 s; Fs = 250 Hz; res = 16 b; 1750 synt. records; 34 channels [69]; T = 300 s; Fs = 1 kHz; res = 16 b; T = 300 s; Fs = 1 kHz; res = 16 b; GA = 38–41 weeks; 5 real records; 5 channels [10,47,48,49,50]; T = 60, 600 and 3600 s; Fs = 1 kHz; res = 12 b; 4 channels; 175 real records [50]
SCBSS [70] Section 2.7	Accurate	Insufficient	MIT-BIH [67]	1 synt. record [70]; T = 0.5 h; Fs = 360 Hz; res = 11 b; 48 real records [67]
TS [45,71] Section 2.8	Moderately accurate	Insufficient	adfecgdb [10,47,48,49,50]	T = 300 s; Fs = 1 kHz; res = 16 b; GA = 38–41 weeks; 5 real records; 5 channels [10,47,48,49,50]
STVD [72] Section 2.9	Accurate	Insufficient	fecgsyndb [69]; ECG physionet challenge 2013 [50]	T = 300 s; Fs = 250 Hz; res = 16 b; 1750 synt. records; 34 channels [69]; T = 60, 600 and 3600 s; Fs = 1 kHz; res = 12 b; 4 channels; 175 real records [50]
EMD [5,73] Section 2.10	Accurate	Insufficient	—	Without tech. specification [5,73]
ICA [74,75] [78,79,80,81,82] Section 3.1	Accurate	Moderately accurate	Katholieke U. Leuven [76]; de Lathauwer [77]; NI-fECG [54,55]; ICALAB toolbox [83]; De-Moor [53]	T = 10 s; Fs = 250 Hz; res = 12 b; 8 real records [76]; T = 300 s; Fs = 500 Hz; 8 channels[79]; T = 300 s; Fs = 500 Hz; 8 channels[79]; T = 60 s; Fs = 500 Hz; res = 12 b; 8 channels [77]; T = 10 s; Fs = 1 kHz; res = 16 b; GA = 21–40 weeks; 55 real records [54,55]; T = 10 s; Fs = 250 Hz [53]
SVD [85] Section 3.2 PCA [88] Section 3.3	Inaccurate Moderately accurate	Insufficient Insufficient	— —	T = 60 s; Fs = 500 Hz; 8 channels [85] T = 10 s; Fs = 500 Hz; 8 real records [88]
πCA [88] Section 3.4	Very accurate	Moderately accurate	—	Fs = 500 Hz; 8 synt. records [88]
SA [89] Section 3.5	Accurate	Moderately accurate	—	20 real records [89]
BA [90,91] Section 3.6	Moderately accurate	Insufficient	De-Moor [53]	T = 10 s; Fs = 250 Hz [53]
ZA [92] Section 3.7	Moderately accurate	Insufficient	De-Moor [53]	4 synt. records [92]; T = 10 s; Fs = 250 Hz [53]
SM [93] Section 3.8	Accurate	Moderately accurate	De-Moor[53]	T = 10 s; Fs = 250 Hz [53]
QIO [94] Section 3.9	Accurate	Moderately accurate	ECG physionet challenge 2013 [50]	T = 60, 600 and 3600 s; Fs = 1 kHz; res = 12 b; 4 channels; 175 real records [50]
PEVD [95] Section 3.10	Accurate	Moderately accurate	MIT-BIH [67]; ECG physionet challenge 2013 [50]	T = 0.5 h; Fs = 360 Hz; res = 11 b; 48 real records [67]; T = 60, 600 and 3600 s; Fs = 1 kHz; res = 12 b; 4 channels; 175 real records [50]
FCM [96] Section 3.11	Accurate	Moderately accurate	—	T = 7 s; Fs = 500 Hz; 2 real records [96]
CS [97] Section 3.12	Accurate	Moderately accurate	adfecgdb [10,47,48,49,50]; ECG physionet challenge 2013 [50]	T = 300 s; Fs = 1 kHz; res = 16 b; GA = 38–41 weeks; 5 real records; 5 channels [10,47,48,49,50]; T = 60, 600 and 3600 s; Fs = 1 kHz; res = 12 b; 4 channels; 175 real records [50]
MCSM [98] Section 3.13	Accurate	Moderately accurate	—	3 real records [98]
πTucker [99] Section 3.14	Moderately accurate	Insufficient	adfecgdb [10,47,48,49,50]	T = 300 s; Fs = 1 kHz; res = 16 b; GA = 38–41 weeks; 5 real records; 5 channels [10,47,48,49,50]
MEMD [100] Section 3.15	Accurate	Moderately accurate	adfecgdb [10,47,48,49,50];	T = 300 s; Fs = 1 kHz; res = 16 b; GA = 38–41 weeks; 5 real records; 5 channels [10,47,48,49,50] 1 real record [101]
ICA-EEMD-WS [102] Section 4.1	Very accurate	Promising	NI-fECG gen. [103]; MIT-BIH [67]; adfecgdb [10,47,48,49,50]	Fs = 1 kHz; 500 synt. records [103]; T = 0.5 h; Fs = 360 Hz; res = 11 b; 48 real records [67]; T = 300 s; Fs = 1 kHz; res = 16 b; GA = 38–41 weeks; 5 real records; 5 channels [10,47,48,49,50]
ICA & AF [104] Section 4.2	Very accurate	Promising	MIT-BIH [67];	T = 0.5 h; Fs = 360 Hz; res = 11 b; 48 real records [67]; T = 10 s; Fs = 250 Hz [53]
ICA & PF [105] Section 4.3	Very accurate	Promising	—	4 real records; 4 channels [105]
π-ICA [107] Section 4.4	Very accurate	Moderately accurate	De-Moor [53]	T = 10 s; Fs = 250 Hz [53]
ICA & PCA [108] Section 4.5	Accurate	Moderately accurate	ECG toolbox [108]; de Lathauwer [77]	8 synt. records; 5000 samples [108]; T = 60 s; Fs = 500 Hz; res = 12 b; 8 channels [77];
ICA & SVD [109] Section 4.6	Accurate	Moderately accurate	—	2 synt. records [109]; T = 600 s; Fs = 300 Hz; 1 real record [109]
BA & ZA [110] Section 4.7	Very accurate	Promising	De-Moor [110]	T = 10 s; Fs = 250 Hz [110]
SVD & PC [111] Section 4.8	Moderately accurate	Insufficient	de Lathauwer [77]	1 synt. record [111]; T = 60 s; Fs = 500 Hz; res = 12 b; 8 channels [77];
PN & SGSF [112] Section 4.9	Accurate	Insufficient	ECG physionet challenge 2013 [50]	1 synt. record [112]; T = 60, 600 and 3600 s; Fs = 1 kHz; res = 12 b; 4 channels; 175 real records [50]
